# Frog Skin Derived Peptides With Potential Protective Effects on Ultraviolet B–Induced Cutaneous Photodamage

**DOI:** 10.3389/fimmu.2021.613365

**Published:** 2021-06-02

**Authors:** Han Liu, Xiaopu Guo, Tangwei Yi, Yihan Zhu, Xinyi Ren, Renxian Guo, Yi Dai, Shaohui Liang

**Affiliations:** School of Basic Medical Sciences, Wenzhou Medical University, Wenzhou, China

**Keywords:** amphibian, skin, peptide, ultraviolet B, reactive oxygen species, inflammation

## Abstract

*Hyla annectans* is a tree frog living in the southwestern plateau area of China where there is strong ultraviolet radiation and long duration of sunshine. So their naked skin may possess chemical defense components that protect it from acute photo-damage. However, no such peptide or components has been identified till to date. In the current work, two novel peptides (FW-1, FWPLI-NH2 and FW-2, FWPMI-NH2) were identified from the skin of the tree frog. Five copies of FW-1 and four copies of FW-2 are encoded by an identical gene and released from the same protein precursor, which possess 167 amino acid residues. FW-1 and -2 can exert significant anti-inflammatory functions by directly inhibiting Ultraviolet B irradiation (UVB)-induced secretion of inflammatory cytokines such as tumor necrosis factor-α (TNF-α) and interleukin-6 (IL-6). They may achieve this function by modulating the UV-induced stress signaling pathways such as Mitogen-activated protein kinases (MAPK) and Nuclear Factor Kappa B (NF-κB). Besides, FW-1 and -2 showed potential antioxidant effects on epidermis by attenuating the UVB-induced reactive oxygen species (ROS) production through an unknown mechanism. Considering small peptides’ easy production, storage, and potential photo-protective activity, FW-1/2 might be exciting leading compounds or templates for the development of novel pharmacological agents for the suppression of UVB-induced skin inflammation. Moreover, this study might expand our knowledge on skin defensive mechanism of tree frog upon UVB irradiation.

## Introduction

Overexposure to ultraviolet rays (UV) radiation is dangerous and has significant harmful effects on human health. A World Health Organization (WHO) report indicated that too much exposure to UV light caused up to 60,000 deaths worldwide with more than 1.5 million disability every year due to excessive UVR exposure (1). It has been proven that ultraviolet B (UVB) irradiation (290-320 nm wavelength) can induce significant alterations in skin, including sunburns, wrinkles, diminished immunity against infections, premature aging and cancer (2–4). UVB irradiation can cause acute inflammatory responses in the skin by inducing the secretion of pro-inflammatory cytokines, such as tumor necrosis factor-α (TNF-α) and interleukin-6 (IL-6), from keratinocytes (5). It’s now believed that UV radiation promotes reactive oxygen species (ROS) accumulation and DNA damage in keratinocytes, which then activate the Nuclear Factor Kappa B (NF-κB) signaling pathway responsible for cytokine release (6).

Among vertebrates, amphibian skin may be the most fragile because they are naked and directly exposed to various stimulus factors from outside living environments such as pathogens, predators, and chemical or mechanistic injuries (7). Therefore, in adaptation to the environment, their skins evolved an excellent chemical defense system composed of a package of pharmacological compounds and gene-encoded peptides/proteins with multiple functions including neurotoxic, analgesic, antimicrobial, and antioxidant activities (8). However, what we’ve discovered is still quite limited and there are many pharmacological components in frog skins wait to be identified and characterized. These pharmacological compounds characterized from amphibian skin will certainly be beneficial for future drug development.

Tree frogs spend a large portion of lifespan on trees than other amphibians, this specific living habits make them more susceptible to various risk factors. So it’s rational to assume that chemical components in their skins may play pivotal roles for maintaining their survival. Some previous studies have reported that there are some pharmacological peptides or proteins with different functions including neurotoxic, analgesic and antimicrobial activities in tree frog skins (9–11). *Hyla annectans* is a tree frog mainly distributed in southwestern plateau (altitude around 2300 m) of China where there is strong ultraviolet radiation and long duration of sunshine. Its skin may possess chemical defense components that protect it from acute photo-damage. To unravel the mystery of its skin chemical defense mechanisms, we purified and characterized two families of immunoregulatory peptides, FW-1 and FW-2, from the skin secretions of the tree frog *Hyla annectans*. FW-1 and -2 were shown to suppress UVB-induced inflammatory reactions by inhibiting cytokine secretion and reactive oxygen species generation.

## Materials and Methods

### Chemicals and Reagents

Antibodies against β-actin, Extracellular signal-regulated kinase (ERK), pERK, c-Jun N-terminal kinase (JNK), pJNK and cleaved caspase 3 were obtained from Cell Signaling Technology (Danvers, MA, USA). Antibodies against p65, p-p65^Ser536^ and IκBα were obtained from Beyotime Biotechnology (Shanghai, China). CM-H2DCFDA was purchased from MedChemExpress (NJ, USA). All other chemicals were purchased from Sigma-Aldrich (Saint Louis, MO, USA) unless otherwise indicated.

### Collection of Frog Skin Secretions and Peptide Purification

**Figure 1:**
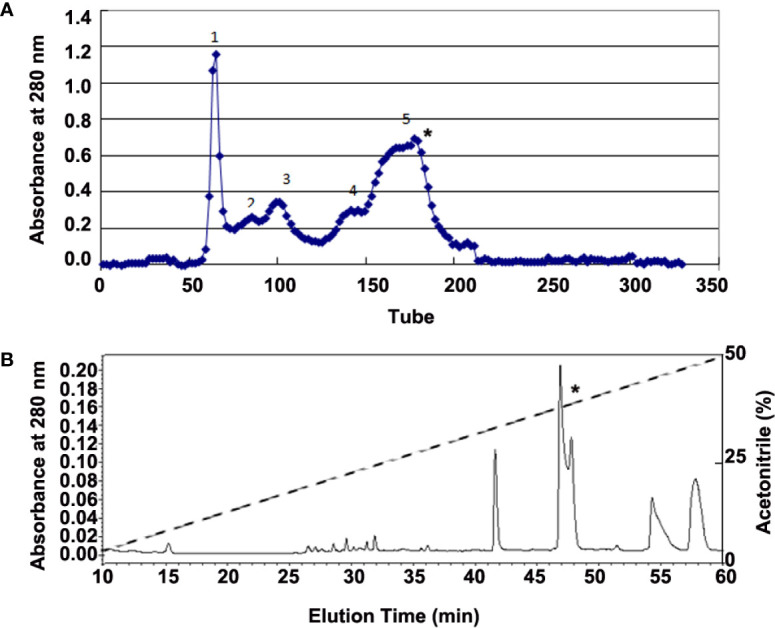
Purification of bioactive peptides from skin secretions of *H. annectans*. **(A)** Lyophilized skin secretion sample of *H. annectans.* was subjected to Sephadex G-75 gel filtration; the elution was performed by 0.1M PBS with a flow rate of 0.3 mL/min. **(B)** Fraction 5 from **(A)** was further purified by RP-HPLC (Hypersil BDS C18, 25×0.46 cm) column. The elution was performed at a flow rate of 0.7 mL/min with gradients of acetonitrile in 0.1% (v/v) trifluoroacetic acid (TFA) in water. The fractions with the interested peptides are marked with stars.

Adult *H. annectans* of both sexes (n=150; weight range 3–5 g) were collected in the Yunnan Province of China. Skin secretions were collected with a 6-volt electronic stimulation for 3–5 s on the skin. Lyophilized skin secretions (4 g, total A_280 nm_ of 1200) were then dissolved in 0.1M NaCl solution. Peptide purification was performed by Sephadex G-75 (Superfine, Amersham Biosciences, 2.6 × 100 cm) gel filtration chromatography and interested fractions were further purified by C_18_ reverse phase high performance liquid chromatography (RP-HPLC, Hypersil BDS C_18_, 30 × 0.46 cm) as illustrated in **Figure 1**.

### SMART cDNA Synthesis and cDNA Cloning

Total RNA of frog skin was extracted using TRIzol (Invitrogen, Ltd.) from a single skin sample of tree frog according to previous literature (9). Briefly, 1 mg skin sample was added to 1 ml pre-chilled TRIzol and got fully homogenized. Then chloroform, isopropanol and ethanol were added respectively to isolate total RNA. cDNA was synthesized using a SMART™ PCR cDNA synthesis kit (Clontech, Palo Alto, CA, USA) according to manufacturer’s instruction. Two oligonucleotide primers, S_1_(5’-CA(A/G)GA(T/C)TA(T/C)AG(A/G)TG(T/C)CA(A/G)(T/C)T(A/G.T/C)TC(A/G.T/C)-3’, in the sense direction, and primer II A provided from the SMART™ PCR cDNA synthesis kit, coupled with specific primers designed according to the amino acid sequence were used for PCR reactions. The DNA polymerase was Advantage polymerase from Clontech. PCR products were cloned into pGEM-T Easy vector (Promega, Madison, WI, USA). DNA sequencing was performed with an Applied Biosystems DNA sequencer, Model ABI PRISM 377.

### Peptides Synthesis

All of the peptides (FW-1/2) used in this work were synthesized by Sangon Biotech (Shanghai) Ltd. (Shanghai, China) and analyzed by HPLC and mass spectrometry to confirmed purity greater than 98% (**Figure S1**). All peptides were dissolved in PBS.

### Cell Culture and Mice

UV irradiation is known to elicit acute skin inflammation by stimulating the secretions of many proinflammatory cytokines in epidermal keratinocytes (5). So we use Human immortalized keratinocyte cells (HaCaT cells) as cell model according to previous literature (12). HaCaT cells were provided by the American Type Culture Collection (ATCC, Manassas, VA), and grown in Dulbecco’s Modified Eagle Medium (DMEM) supplemented with 10% fetal bovine serum (FBS), 100 U/mL penicillin and 100 μg/mL streptomycin. All cell culture was maintained in a humidified incubator with 5% CO_2_ at 37°C. All chemical reagents including the medium and serum used in cell culture experiment were purchased from GIBCO (ThermoFisher, Waltham, MA, USA).

C57BL/6 mice (SPF, male, wild-type, aged 8 weeks, 22–24 g) were used for all experiments. Because female mice may be easily affected by hormone, we use male in this study. All mice were housed and kept in animal facility at the Wenzhou Medical University under standard conditions (22 ± 2°C, 40% ~ 60% humidity, 12-h light and 12-h dark cycle). The dorsal skin was shaved and chemically depilated by Veet cream 48 h before UV irradiation.

### UVB Irradiation

All UV irradiations were performed as previously published (13, 14). Briefly, the HaCaT cells and mice were exposed to a spectral peak on 312 nm of the UVB irradiation with NB-UVB system at a single dose (10 mJ/cm^2^ for cells and 500 mJ/cm^2^ for mice). Before the UVB irradiation, cells were pre-treated without and with different concentrations (6, 12, and 24 μg/ml) of FW-1, FW-2 for 30 min. During the period of the experiment, a part of samples was taken from each treatment for analysis at the indicated time points. Three replications were used for each treatment. For *in vivo* exposures, mice with their back skin chemically depilated 48 hr before exposures, were intradermally injected with FW-1 and -2 (5 mg/kg) and simultaneously subjected to a single dose of UVB irradiation. The concentrations were selected and tested according to previous literatures (15).

### Measurement of ROS Generation

The fluorometric assay (DCFH-DA assay) was used to measure the intracellular ROS level according to previous literature (14). HaCaT cells seeded in 96-well microplates were pre-treated with 5 mM N-acetyl cysteine (NAC) or FW1, FW2 with indicated concentration for 1 h. Then the cells were incubated for 30 min after the addition of 10 μM H2DCFDA and exposed to 20 mJ/cm^2^ UVB, followed by lysis with 0.1% Triton X-100 for 10 min. The fluorescence intensity of was measured with an excitation of 485 nm and emission at 528 nm using a Varioskan LUX multimode microplate reader (ThermoFisher, Waltham, MA, USA). Intracellular ROS levels are expressed as the fold change of the fluorescence intensity.

### Pro-inflammatory Cytokine Determination

HaCaTs were co-incubated with FW-1/2 (0, 6, 12, and 24 μg/ml) for 1 hr before UV (10 mJ/cm^2^) irradiation as described above. After 6 h, the cells and the cell culture supernatants were collected for quantitative real-time PCR (qRT-PCR) and Enzyme linked immunosorbent assay (ELISA) (eBiosciences) to examine the mRNA and protein level of pro-inflammatory cytokines TNF-α and IL-6.

The effects of FW-1 and -2 on UV-induced pro-inflammatory cytokine production in mice were also determined. Skin tissues were obtained from mice after 48 h of UV irradiation (500 mJ/cm^2^). Levels of protein for mouse IL-6 and TNF-α were determined by ELISA.

### Histology and Immunofluorescence Analysis

Skin samples of mice were fixed in 4% paraformaldehyde, embedded in optimal cutting temperature (OCT) compound, frozen, and sectioned at 10 μm. Sections were subjected to hematoxylin and eosin staining or immunofluorescence staining as described previously (16). Antibodies were diluted according to the instructions of the manufacturer unless indicated otherwise.

### Statistical Analysis

Statistical analysis was evaluated using GraphPad Prism 8 (GraphPad Software, La Jolla, CA, USA) by one-way ANOVA unless otherwise indicated. A P-value of less than 0.05 was considered as statistically significant. All results are expressed as the mean ± SD.

## Results

### Purification of Bioactive Peptides from the Frog Skin Secretions

Considering that UV irradiation stimulates the secretions of many pro-inflammatory cytokines such as IL-6 and TNF-α in epidermal keratinocytes (5), we used IL-6 as a marker for identifying the possible anti-inflammatory peptides from all purified fractions. The supernatant of *H. annectans* skin secretions was divided into five fractions after Sephadex G-75 gel filtration, and the fraction 5 indicated by a star was found to inhibit UVB-induced HaCaT cells IL-6 secretion as illustrated in **Figure 1A**. The fraction with IL-6 secretion inhibitory activity was concentrated and further purified by C18 RP-HPLC. Two peptides were purified from this step as illustrated in **Figure 1B** in a fraction with a star, which are named FW-1 and -2, respectively. The complete amino acid sequences by Edman degradation of FW-1 and -2 were determined as FWPLI (FW-1) and FWPMI (FW-2), respectively. It’s well known that carboxypeptidase Y treatment will release free amino acids from a peptide with free C-terminal -COOH group. However, treating both FW-1 and -2 with carboxypeptidase Y did not lead to the release of free amino acids, indicating that C-terminal ends of FW-1 and -2 are amidated, which gave the calculated molecular masses of 673.8 and 691.9, respectively. This is well matched with MALDI-TOF-MS analysis of target RP-HPLC fraction, which gave observed molecular masses of 674.5 and 691.5 (**Figure S2**), respectively. Therefore, the amino acid sequence of FW-1 and -2 is FWPLI-NH2 and FWPMI-NH2, respectively. The amino acid sequences of FW-1 and -2 were further confirmed by the cDNA cloning as described below.

### cDNA Cloning

**Figure 2:**
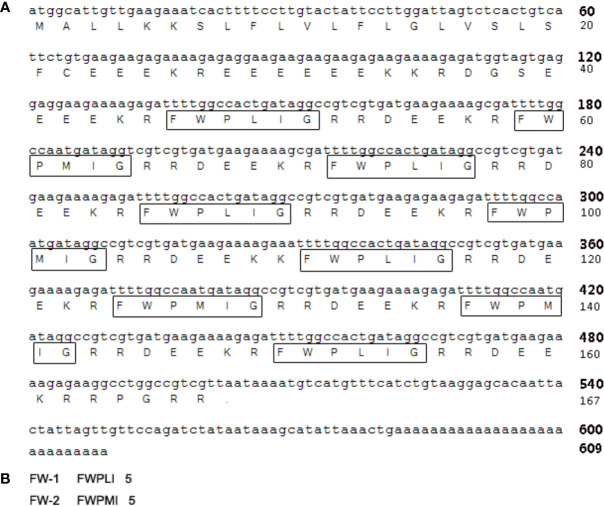
The nucleotide sequence encoding FW-1/2 precursor and the amino acid sequence. **(A)** The cDNA sequence encoding FW-1 and FW-2 from the skin of tree frog *H. annectans*. The mature FW-1 and -2 are boxed. **(B)** The amino acid sequences of mature FW-1 and -2.

As illustrated in **Figure 2A**, a cDNA of 609 bp (GenBank™ accession MT036977) was cloned from the cDNA library of skin of tree frog *H. annectans*. This cDNA encodes a precursor protein composed of 167 amino acid residues. Both FW-1 and -2 share the same 167-amino acid precursor protein. In the sequences of this precursor, there are five copies of FW-1 and four copies of FW-2 (**Figure 2B**). Both FW-1 and -2 are composed of 5 amino acid residues, which are in consistent with the Edman degradation sequences from the purification. There are bibasic enzymatic processing sites (-KR- and -RR-) flanked at N- and C-terminus of these mature peptides, respectively. (**Figure 2A**). This precursor share high similarity with both analgesin precursor including 12 copies of analgesin (FLPFL) found in the skin of the tree frog, *H. simplex* (9) and analgesin precursor with 10 copies of analgesin (FWPVI and FWPVT) from the skin of the tree frog, *H. japonica* (11).

### Effects on UVB-Induced Skin Inflammation

**Figure 3:**
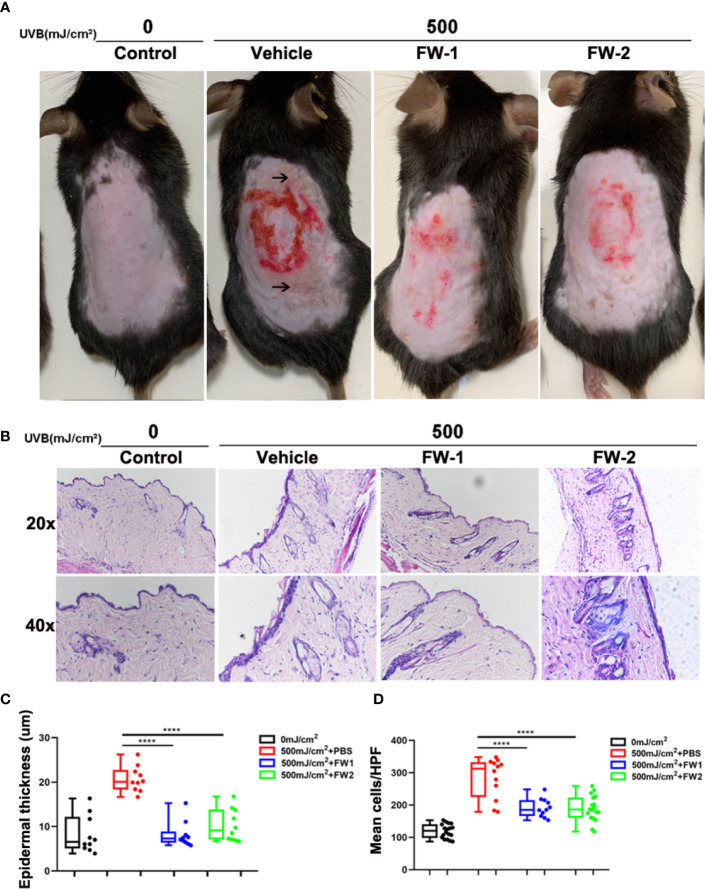
FW-1 and -2 have protective effects in UV-induced skin inflammation. **(A)** Representative photographs of mice skin with or without FW-1 and -2 treatment (5 mg/kg) after 48 hr post UV exposure (500 mJ/cm^2^ UVB). Arrows indicate areas with obvious sunburn reactions such as blisters and skin peeling. Red circle indicate areas where we intradermal injected with samples and section for HE staining. **(B)** Histology of the mice skin with or without FW-1 and -2 treatment after 48 hr of UV irradiation (500 mJ/cm^2^ UVB). Skin sections were stained with H&E. Box and whisker plot indicating skin thickness **(C)** and immune cell infiltration **(D)** measured after 48 hr post UV irradiation (500 mJ/cm^2^ UVB) using ImageJ software (n=10). Scale bars, 50 μm. ****P < 0.0001.

UV irradiation can cause dramatic alteration in the skin composition including erythema, edema, epidermal hyperplasia and infiltration of immune cells, all of which are characteristic of a sunburn reaction (17–19). To determine whether FW-1 and -2 have any protective effects in UV-induced skin inflammation, we compared the effects of UV irradiation on the skin of mice with intracutaneous application of FW-1 and -2 (5 mg/kg). As illustrated in **Figure 3A**, UVB irradiation promoted a significant sunburn reaction including edema, blisters and skin peeling. While mice treated with FW-1 and -2 showed smoother skins and a much more alleviated sunburn symptoms. After 48 h of UVB irradiation, FW-1 and -2 treated mice showed significantly decreased swelling of the skin (**Figures 3B, C**) and reduced infiltration of immune cells to the UV-irradiated skin (**Figures 3B, D**), compared with those of PBS vehicle treatment control. These skin phenotypes of mice suggest a protective role of FW-1 and -2 in UV-induced acute inflammation.

### Effects on UVB-Induced Inflammatory Cytokines Production in Keratinocytes

**Figure 4:**
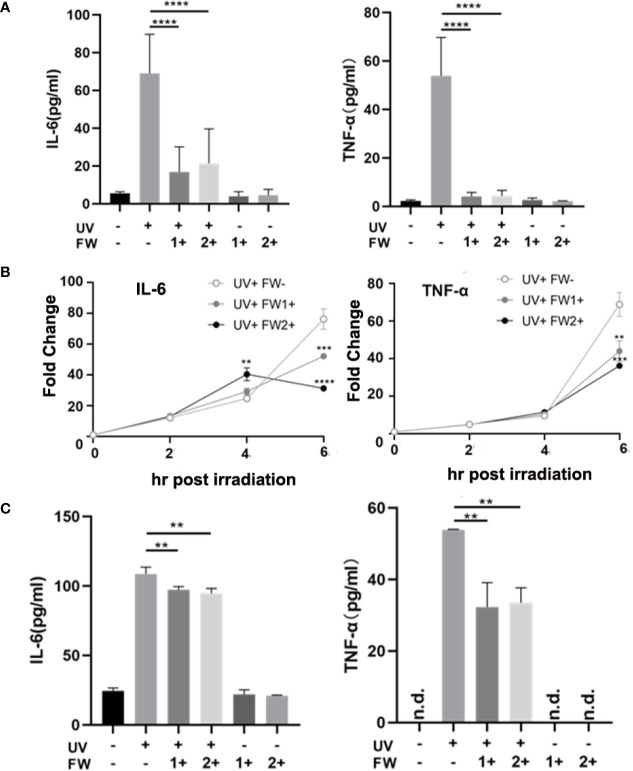
FW-1 and -2 suppress the expression of inflammatory cytokines in UV-irradiated keratinocytes. **(A)** Skin tissues of mice with or without FW-1 and -2 treatment (5 mg/kg) were obtained after 48 hr post UV exposure (500 mJ/cm^2^ UVB). Mouse IL-6 and TNF-α expression level were determined by ELISA (n=5). ****P < 0.0001. HaCaT cells with or without FW-1 and -2 (12 μg/ml) pre-treatment were exposed to UV irradiation (10 mJ/cm^2^). After 6 hr, levels of mRNA **(B)** and secreted protein **(C)** for TNF-α and IL-6 were measured by QRT-PCR and by ELISA, respectively. (n=3). Error bars represents mean ± S.D. **P < 0.01, ***P < 0.001, ****P < 0.0001.

UV irradiation is known to elicit acute skin inflammation by stimulating the secretions of many proinflammatory cytokines such as IL-6 and TNF-α in epidermal keratinocytes (5). To evaluate whether FW-1 and -2 have any effects on UVB-induced cytokines production, we test the effects of FW-1 and -2 on producing inflammatory cytokines in response to UVB irradiation in mice. As shown in **Figure 4A**, the protein levels of both TNF-α and IL-6 were significantly elevated in UV-exposed mice skin, while FW-1 and -2 treatment dramatically inhibited the secretion of both cytokines compared to the vehicle-treated control group in response to UVB irradiation.

To test the effects of FW-1 and -2 in human keratinocytes, we compared the levels of IL-6 and TNF-α expression in keratinocytes with or without FW-1 and -2 treatments in response to UVB irradiation. We’ve tried three concentrations of FW-1 and -2 (6, 12, and 24 μg/ml), and found that 12 μg/ml is the optimal concentration with the best effects. So this concentration was used in all the following experiments unless otherwise indicated. As shown in **Figure 4B**, the mRNA levels of TNF-α and IL-6 in untreated control HaCaT cells rapidly increased after UV irradiation, while the mRNA levels of both cytokines in FW-1 and -2 treated cells were suppressed in response to UVB irradiation. ELISA results showed that the protein levels of TNF-α and IL-6 secreted from UV-irradiated HaCaT cells were also reduced when pre-treated with FW-1 and -2 (**Figure 4C**). These results indicate that FW-1 and -2 have a potential anti-inflammatory effect in skin through inhibiting some pro-inflammatory cytokines production in epidermal keratinocytes when exposed to UVB irradiation.

### Effects on UVB-Induced ROS Production

**Figure 5:**
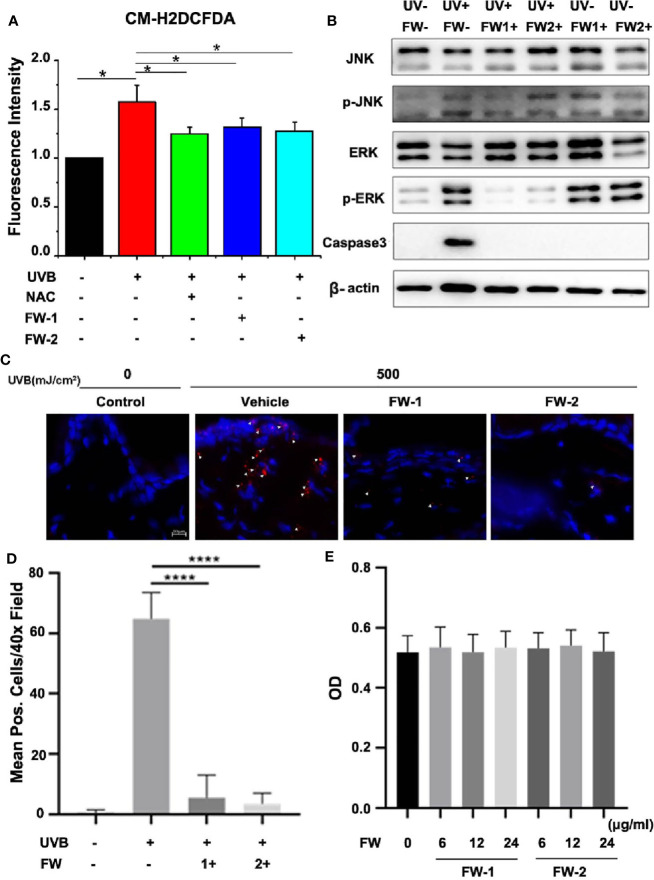
FW-1 and -2 inhibit ROS accumulation in UV-irradiated keratinocytes. **(A)** HaCaT cells with or without FW-1 and -2 (12 μg/ml) pre-treatment were irradiated with 10 mJ/cm^2^ UVB. FW-1 and -2 or positive control NAC (5 mM) was added 1 hr before UVB exposure. After irradiation, ROS levels were measured using a microplate reader (n=3). Error bars represents mean ± S.D. *P < 0.05. **(B)** HaCaT cells with or without FW-1 and -2 (12 μg/ml) pre-treatment were exposed to UVB radiation (10 mJ/cm^2^) and incubated for 4 hr. The cell lysates were then immunoblotted with the indicated antibodies. **(C)** Skin tissues of mice with or without FW-1 and -2 treatment were obtained after 48 hr post UV exposure (500 mJ/cm^2^ UVB). Skins were sectioned and processed for anti-cleaved caspase 3 immunostaining (red). The arrowheads mark the positively stained cells. The blue signals indicate nuclear staining. Scale bars, 10 μm. **(D)** Quantification of stained cells per high power field from skins in **(C)** ****P < 0.0001. **(E)** HaCaT cells treated with FW-1 and -2 at different doses relevant to our studies were exposed to UVB radiation (10 mJ/cm^2^) and incubated for 18 hr later for cytotoxicity analysis using MTT Cell Proliferation and Cytotoxicity Assay Kit. No significant differences were detected between groups (n=3). P > 0.1.

Many previous studies have shown that there is a significant elevation and accumulation of ROS in skin after UV irradiation, which will trigger skin cell damage and accelerate skin aging and skin cancer (20, 21). Mechanistically, ROS and DNA damage in keratinocytes caused by UV irradiation is reported to trigger cytokine release and responsible for skin inflammation (6, 22). To reveal whether FW-1 and -2 have protective effects on epidermal keratinocytes upon UVB irradiation, we tested the effects of synthesized FW-1 and -2 on UVB-induced ROS accumulation. Consistent with the previous reports (21, 23), we found that ROS production significantly increased when HaCat cells were irradiated by UVB (**Figure 5A**). We also used N-acetyl cysteine (NAC), a widely used antioxidant as the positive control, and confirmed that it significantly attenuated the UVB-induced ROS production (**Figure 5A**). As illustrated in **Figure 5**, both FW-1 and -2 could markedly inhibit production of UVB-induced ROS at the concentration of 12 μg/ml, which showed the effects as good as the positive control NAC. Previous studies have indicated that UV irradiation will cause skin cell damage and lead to intracellular ROS accumulation (20, 21). While NAC can significantly attenuated the UVB-induced ROS production in the sunburn keratinocytes by enhancing apoptosis of the damaged cells (14). To reveal whether FW-1 and -2 have the same protective mechanism as NAC, we measured the expression of cleaved form of caspase 3 to detect UV-induced keratinocyte cell death. As expected, UV irradiation caused a significant increased expression of cleaved caspase 3 in skin epidermis. Interestingly, mice treated with both FW-1 and -2 showed a dramatic inhibitory effects on its expression, almost to the extend of the un-irradiated control level (**Figure 5B**). Next, we performed IF staining of skin specimens and observed a significant increase in epidermal keratinocytes positive for cleaved caspase 3 upon UV irradiation while FW-1 and -2 treatment dramatically reversed this effects (**Figure 5C**), which is in consistent with the previous western blot analysis. This finding indicates that FW-1 and -2 may execute an antioxidant mechanism completely different with the NAC, not by inducing apoptosis of the damaged keratinocytes, but by protecting the keratinocytes from damage upon UV irradiation. Besides, FW-1 and -2 alone did not display any epidermal cytotoxic effects (**Figure 5C**). Likewise, both FW-1 and -2 treatments in cell culture did not display toxicity at doses relevant to our studies (**Figure 5D**). Taken together, FW-1 and -2 showed a potential antioxidant effects on epidermis by attenuating the UVB-induced ROS production through a protective mechanism differently from most of widely used antioxidants such as NAC.

### Effects on UVB-Induced Stress Signaling

**Figure 6:**
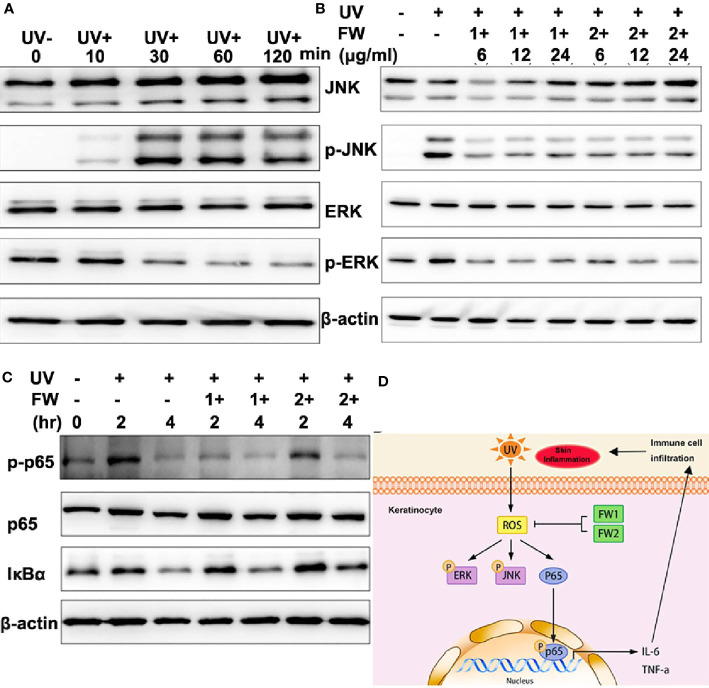
FW-1 and -2 suppress UV-induced stress signaling in keratinocytes. **(A)** HaCaT cells were exposed to UVB radiation (10 mJ/cm^2^) and incubated for the indicated times. The cell lysates were then immunoblotted with the indicated antibodies. **(B)** HaCaT cells treated with FW-1 and -2 at different doses relevant to our studies were exposed to UV irradiation (10 mJ/cm^2^). After 4 hr, the extracted proteins were then immunoblotted with the indicated antibodies. **(C)** HaCaT cells with or without FW-1 and -2 treatment were exposed to UVB radiation (10 mJ/cm^2^) and incubated for the indicated times. The cell lysates were then immunoblotted with the indicated antibodies. **(D)** Schematic representation of frog skin derived peptides FW-1 and -2 act as modulator in UV-induced inflammatory signaling.

It’s well known that UVB can activate several signaling pathways such as hypoxia-inducible factor (HIF), the p53, and mitogen-activated protein kinase (MAPK) signaling pathways. As a highly conserved serine/threonine protein kinases family, MAPK is essentially participated in a bunch of fundamental cellular processes such as proliferation, differentiation, apoptosis, and stress response. Previous studies have reported that UVB irradiation can lead to epidermal growth factor receptor (EGFR) phosphorylation, which then activates ERK1/2 and JNK MAPK pathways in keratinocytes (24–26). These reports also point out that UVB induced ROS can act as mediators to activate MAPK (24, 27). As we have observed that both FW-1 and -2 could markedly inhibit production of UVB-induced ROS at a low concentration, we would like to determine the effects of FW-1/2 on JNK and ERK1/2 phosphorylation in keratinocytes. As shown in **Figure 6A**, the level of JNK phosphorylation in HaCat cells started to increase significantly in the 30 mins immediately after UVB irradiation and gradually decreased within 2 hrs post irradiation. Phosphorylation of ERK1/2, on the other hand, was first significantly decreased at 30 mins and subsequently kept this decreased level till 2 hr after irradiation as previously reported (28). These results indicate that UVB activates JNK and ERK1/2 MAPK in HaCats. In addition, in the present of FW-1/2, ERK1/2 and JNK phosphorylation induced by UVB were significantly inhibited at doses relevant to our studies (**Figure 6B**), which is consistent with Western blot analysis of epidermal lysates (**Figure 5B**), suggesting that FW-1/2 made an impact on the MAPK signaling pathway in response to UVB irradiation.

UVB irradiation has been reported to cause acute inflammatory responses in the skin by inducing the generation of ROS and DNA damage in keratinocytes, which then activate the NF-κB signaling pathway responsible for pro-inflammatory cytokine secretion (2, 6). To determine whether FW-1 and -2 have any effect on NF-κB activation under UV irradiation, we monitored the protein levels of IκBα and phosphorylated p65. As illustrated in **Figure 6C**, IκBα levels were decreased after UV irradiation in a time-dependent manner and there were no significant difference between FW-1 and -2 treated and untreated group. By contrast, the levels of phosphorylated p65 (p-p65^Ser536^) were dramatically increased within 2 hrs post UV irradiation and fallen back to the basal level till 4 hr in control group, but was significantly inhibited by FW-1 and -2 treatment at 2 hr post irradiation. Besides, FW-1 seems to exhibit much stronger inhibitory effect than FW-2. This result indicates that FW-1 and -2 modulate NF-κB activation not by affecting IκBα degradation, but by inhibiting the phosphorylation of p65 at Ser^536^.

Taken together, the above results indicate that FW-1 and -2 are the first peptides ever found in tree frog skin with potential anti-inflammatory and photo-damage protective abilities. In addition, both peptides showed antioxidant activities that could inhibit the ROS accumulation induced by UV radiation, also significantly inhibited ERK and JNK MAPK and NF-κB signaling by down-regulating p65 phosphorylation, leading to a decrease in production of pro-inflammatory cytokine such as IL-6 and TNF-α (**Figure 6D**).

## Discussion

Amphibian skin acts as the first defensive barrier against various injuries for their survival. In adaptation to the complex living environment, they have evolved a chemical defense system, which is actually a package of pharmacological compounds and gene-encoded peptides/proteins. The diversity of pharmacological compounds in amphibian skin secretions is surprisingly high, even within a single species, including peptides or proteins with different functions such as neurotoxic, analgesic, antimicrobial, and antioxidant activities (7, 8, 10, 29–33). Tree frogs spend a large portion of lifespan on trees than other amphibians, this specific living habits make them more susceptible to various risk factors such as predator attacking, pathogen infection, and noxious abiotic factors such as UV radiation. Our previous work has identified a number of peptides or proteins with various defensive functions, from skin secretions of the tree frog, *H. simplex* (9). Most of those components can help defend against predators, heal wounds, or attenuate suffering. Among these components, we have identified two novel peptides named analgesin a1 (FLPFL-NH_2_) and a2 (FLPIL-NH_2_) from the skin secretions and these peptides showed strong antinociceptive ability. Similarly, another two novel analgesic peptides (Analgesin-HJ, FWPVI-NH2 and Analgesin-HJ(I5T), FWPVT-NH2) were also identified from the skin of another tree frog, *Hyla japonica* (11). These analgesic peptides are able to block pain and lighten suffering for self-protection.

*Hyla annectans* is a tree frog mainly distributed in southwestern plateau (altitude around 2300 m) of China where there is strong ultraviolet radiation and long duration of sunshine. You et al. has identified the first gene-encoded amphibian neurotoxin anntoxin from the skin secretions of this tree frog (10). Anntoxin is a Kunitz-type protein toxin that markedly inhibits the neuronal TTX-S voltage-gated sodium channel (VGSC). Anntoxin was proved to have lethal toxicity for several potential predators of tree frog, including the insect, snake, bird, and mouse. However, apart from anntoxin, no other peptides or proteins has ever been identified from this species. Liu et al. have identified an antioxidant peptide named antioxidin-RL from frog skin of *Odorrana livida* (31). Similarly, a large amount of antioxidant peptides has been isolated from the skin secretions of *Rana pleuraden*, a frog distributed in the subtropical plateau, southwestern of China (33). These peptides showed rapid free radical scavenging activity with the ABTS^+^ radical scavenging assay. Considering the same specific living environment shared with *R. pleuraden*, we highly suspect that the skin of *Hyla annectans* also has components that protect it from strong ultraviolet radiation and acute photo-damage. However, in contrast to the complex compounds and high diversity of other amphibian skin secretions, the constituents in the skin secretions of *H. annectans* is quite simple and single. So it is rational to hypothesize that the relatively single components in the skin secretions of *H. annectans* may exert multi-functions to make this tree frog adapted to the complex and varied living environments.

The current work identified two novel antioxidant peptides (FW-1 and -2) from the skin secretions of the tree frog *H. annectans*. Both composed of only five amino acid residues with C-terminal ends amidated. There are two (40%) aromatic amino acid residues (F and W) in their sequences. Interestingly, the precursor encoding contains five copies of FW-1 mature peptide and four copies of FW-2 mature peptide (**Figure 2**), which may facilitate to express this peptide with high efficiency in the tree frog skin. Actually, we indeed observed that peptides FW-1 and -2 hold a large proportion of components in the relatively single constituents skin secretions of this tree frog. The skin of *H. annectans* has long been used as poultice in ancient China, as in traditional Chinese medical book it is believed to have wound-healing promoting and analgesic effects. So far, many amphibian-derived peptides that could promote skin wound healing have been characterized with different mechanisms. For example, a wound healing-promoting peptide named Ot-WHP, has been characterized from Chinese concave-eared frog *Odorrana tormota*. It efficiently promoted wound healing in a mouse model of full-thickness wounds by recruiting neutrophils and promoting keratinocyte migration (34). Similar wound-healing promoting peptides have been characterized in other amphibians (35). So it’s rational to assume that *H. annectans* may also has such wound-healing promoting peptides. Besides, as the precursor of FW-1/2 share high similarity with both analgesin precursors found in the skin of the frog, *H. simplex* (9) and *H. japonica* (11), we assumed that FW-1 and -2 also had analgesic activities.

UV irradiation can lead to a series of alteration and responses in the skin, especially inflammatory responses, which contribute to the development of photoaging and even skin cancer (22). UVB irradiation can cause acute inflammatory responses in the skin by inducing the secretion of pro-inflammatory cytokines, such as tumor necrosis factor-α (TNF-α) and IL-6, from keratinocytes (5). It’s now believed that UV radiation promotes reactive oxygen species (ROS) accumulation and DNA damage in keratinocytes, which then activate the NF-κB signaling pathway responsible for cytokine release (6). Previous studies have reported a large amount of antioxidant peptides from the skin secretions of different species of frogs, revealing a new skin antioxidant system (31, 33). All these antioxidant peptides showed excellent free radical scavenging activities and could rapidly scavenge oxidants *in vitro* within several seconds. However, no peptide that could suppress the generation of ROS in keratinocytes has ever been identified. To the best of our knowledge, FW-1 and -2 are the first peptides ever found in amphibian skin with potential antioxidant activities that could inhibit the accumulation of ROS induced by UV radiation in keratinocytes. In addition, both peptides also showed anti-inflammatory and photo-damage protective abilities. As there are multiple evidences of cross-talk between inflammation and oxidants (36), the antioxidant activities of FW-1/2 may contribute to their anti-inflammatory abilities. Besides, FW-1/2’s antioxidant activities against radiation injuries and the relative high concentration in its skin make it an excellent pharmacological component in its skin chemical defense system and enable the forest-living frog to evolve an ecological adaptation.

In summary, the current work identified two multi-functional peptides with both antioxidant and anti-inflammatory activities from the tree frog *H. annectans*. FW-1 and -2 are the first peptide ever found in amphibian skin with potential antioxidant activities that could inhibit the accumulation of ROS induced by UV radiation in keratinocytes. In addition, FW-1/2 has a simple structure with only five amino acids, which is easy for production, store and ship. It might be an excellent leading compound or template for the development of novel antioxidant agent for UV radiation-related skin disorders, such as photoaging and skin cancer, which occur with chronic exposure to UV irradiation. Besides, these findings suggest that the chemical components from tree frog skin might be a useful strategy for the forest-living frog to evolve an ecological adaptation to the radiation injuries. Further work is necessary to investigate the signaling pathways underlying its antioxidant and anti-inflammatory functions.

## Data Availability Statement

The datasets presented in this study can be found in online repositories. The names of the repository/repositories and accession number(s) can be found in the article/**Supplementary Material**.

## Ethics Statement

The animal study was reviewed and approved by the Animal Care and Use Committee of Wenzhou Medical University.

## Author Contributions

HL: Conceptualization, methodology, investigation, writing manuscript, and funding acquisition. XG: Investigation, validation, software, and data analysis. TY and YZ: Investigation. XR, RG, and YD: Data analysis. SL: Project administration and funding acquisition. All authors contributed to the article and approved the submitted version.

## Funding

This work was supported by Chinese National Natural Science Foundation (31801173) and Zhejiang Provincial Natural Science Foundation (LGD21C040009).

## Conflict of Interest

The authors declare that the research was conducted in the absence of any commercial or financial relationships that could be construed as a potential conflict of interest.

## Abbreviations

UVB, Ultraviolet B irradiation; ROS, Reactive oxygen species; TNF-α, Tumor necrosis factor-α; IL-6, Interleukin-6; DMEM, Dulbecco’s Modified Eagle’s Medium; RP-HPLC, Reversed phase high pressure liquid chromatography; ELISA, Enzyme linked immunosorbent assay; MAPK, Mitogen-activated protein kinases; ERK, Extracellular signal-regulated kinase; JNK, c-Jun N-terminal kinase; NF-κB, Nuclear Factor Kappa B NAC, N-acetyl cysteine.
