# Supplementary material for: Frog Skin Derived Peptides With Potential Protective Effects on Ultraviolet B–Induced Cutaneous Photodamage
Source: Front Immunol. 2021 Jun 2;12:613365. doi: 10.3389/fimmu.2021.613365 (PMC8206783; doi:10.3389/fimmu.2021.613365)
Supplement: Supplementary file 1 [file DataSheet_1.docx]

**Frog skin derived peptides with potential protective effects on ultraviolet B–induced cutaneous photodamage**

Han Liu^1, 2^*, Xiaopu Guo^1, 2^, Tangwei Yi^1^, Yihan Zhu^1^, Xinyi Ren^1^, Renxian Guo^1^, Yi Dai^1^ and Shaohui Liang^1^*

1. School of Basic Medical Sciences, Wenzhou Medical University, Wenzhou, Zhejiang, 325035, China.
2. These authors contributed equally to this article.

*To whom correspondence should be addressed

**Supplemental figures**

**
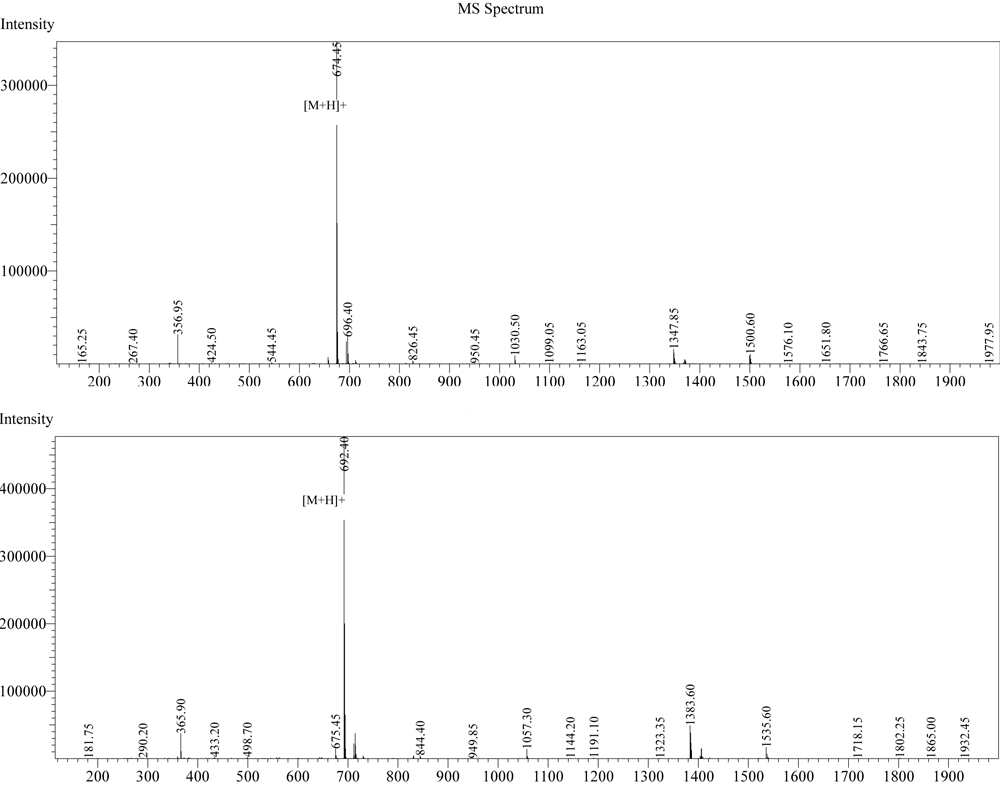
**

**Figure S1.** **MALDI-TOF-MS analysis of synthetic FW-1 and FW-2.**

**
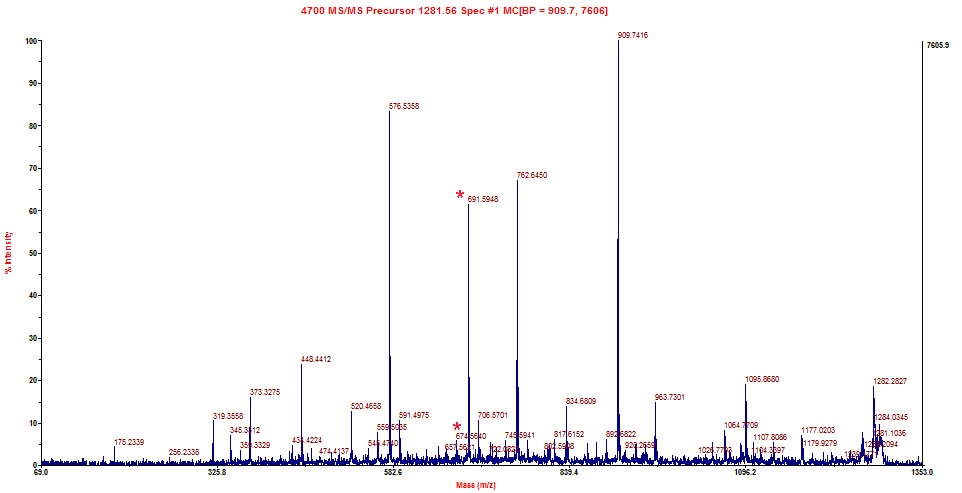
**

**Figure S2. MALDI-TOF-MS analysis of the RP-HPLC target fraction with FW-1 and FW-2 which are marked with star.**
